# Correction of Spatial Bias in Oligonucleotide Array Data

**DOI:** 10.1155/2013/167915

**Published:** 2013-03-13

**Authors:** Philippe Serhal, Sébastien Lemieux

**Affiliations:** ^1^Institute for Research in Immunology and Cancer (IRIC), Université de Montréal, C.P. 6128, Succursale Centre-Ville, Montréal, QC, Canada H3C 3J7; ^2^Department of Computer Science and Operations Research, Université de Montréal, C.P. 6128, Succursale Centre-Ville, Montréal, QC, Canada H3C 3J7

## Abstract

*Background*. Oligonucleotide microarrays allow for high-throughput gene expression profiling assays. The technology relies on the fundamental assumption that observed hybridization signal intensities (HSIs) for each intended target, on average, correlate with their target's true concentration in the sample. However, systematic, nonbiological variation from several sources undermines this hypothesis. Background hybridization signal has been previously identified as one such important source, one manifestation of which appears in the form of spatial autocorrelation. *Results*. We propose an algorithm, *pyn*, for the elimination of spatial autocorrelation in HSIs, exploiting the duality of desirable mutual information shared by probes in a common probe set and undesirable mutual information shared by spatially proximate probes. We show that this correction procedure reduces spatial autocorrelation in HSIs; increases HSI reproducibility across replicate arrays; increases differentially expressed gene detection power; and performs better than previously published methods. *Conclusions*. The proposed algorithm increases both precision and accuracy, while requiring virtually no changes to users' current analysis pipelines: the correction consists merely of a transformation of raw HSIs (e.g., CEL files for Affymetrix arrays). A free, open-source implementation is provided as an R package, compatible with standard Bioconductor tools. The approach may also be tailored to other platform types and other sources of bias.

## 1. Background

Microarray technology, a fairly recent yet already well-established and extensively dissected method, allows for the simultaneous quantification of expression levels of entire genomes or subsets thereof [1]. *In situ* oligonucleotide arrays are by far the most popular type, representing at the time of writing 70% of all arrays deposited in the Gene Expression Omnibus (GEO), a public microarray database, in the last year; of these, 58% are Affymetrix GeneChips [2]. These are designed such that each gene is targeted by multiple perfectly complementary oligonucleotide probes at various locations along its sequence (forming a *probe set*); copies of each of these probes are covalently linked to a solid surface at a predetermined location on a grid; a labelled RNA sample is allowed to hybridize to each of these probes; and finally a hybridization signal intensity (HSI) is obtained for each probe [3]. The technology relies on the assumption that, on average, HSIs observed in a given probe set correlate with the true concentration of the given mRNA species in the biological sample, that is, the true expression level of the targeted gene. Variations on this architecture exist; for example, tiling arrays, are designed such that probes target contiguous regions of a genome, usually without regard for transcript annotations [4].

Because the objective of such experiments is generally to assess gene expression differences between one or more biological samples, separating *biologically interesting* variation from all other sources of *obscuring* variation is of utmost importance [5]; consequently, this has been a major focus of microarray research in the last decade. Whereas random error (i.e., noise) can be estimated via sample variance and cancelled out by some form of averaging, systematic errors introduce biases in the data that cannot be estimated without an independent source of information and cannot be explicitly corrected for without being estimated [6]. As has been shown repeatedly, there are several important sources of systematic errors—notably arising from RNA sample preparation [7]; probe-target binding efficiency [8], specificity [9], and spatial uniformity [10–14]; secondary structure in probes [15] and transcripts [16], and other thermodynamic properties [17] such as GC content [18]; scanner calibration [19]; and algorithmic processing of raw image data [20]—and underestimating their effect on analyses leads to tangible consequences [21].

An initial attempt to address nonuniform “background intensity” was incorporated directly in the design of the GeneChip platform: for each “perfect match” (PM) probe, there is a corresponding “mismatch” (MM) probe which features a single different base [3]. The intention was twofold: to correct for specificity biases, by assuming each PM/MM pair would share nonspecific hybridization signal, while only the PM probe would exhibit specific signal, and to correct for spatial biases, by making each PM/MM pair physically adjacent on the array. In practice, MM probes contain significant specific signal and do not share common nonspecific background with their respective PM probes; in fact, early studies found that approximately one-third of MM intensities are greater than their PM counterpart [22, 23], which is evidently incompatible with their stated purpose. In recent years, MM intensities have largely been ignored, and recent array designs by Affymetrix do not include them [24]. Current popular methods make no attempt to correct for either of these biases.

In order to make data from multiple arrays directly comparable, normalization methods such as locally weighted scatterplot smoothing (LOWESS or loess) [25–28] and Bolstad et al.'s quantile normalization algorithm [27] have been proposed and are currently widely regarded as essential preprocessing steps. The former modifies the HSIs such that a log HSI ratio versus mean log HSI plot becomes locally linear, while the latter forces the HSIs from each array in the experiment to follow the same distribution. It is important to note, then, that neither of these methods attempts to correct for any specific source of obscuring variation, but rather they make a general attempt to craft the raw data from separate arrays such that they become more directly comparable, inevitably discarding information in the process.

It has been noted that the choice of background correction methodology has a significant impact on downstream analysis accuracy [29], which implies that nonspecific hybridization and spatial nonuniformity should not be ignored. We focus here exclusively on the latter; the reader is referred to [30–32] for treatments of the former. A few methods addressing spatial biases in spotted cDNA arrays have been proposed. Dudoit et al. proposed the only such method to gain wide acceptance in the community, which consists of applying the loess-based intensity-dependent bias correction method individually within each print-tip group [25, 26, 33]. Colantuoni et al. proposed to subtract a 2D loess fit of intensities on the array surface [34], while Workman et al. similarly subtract an estimate of local bias based on a 2D weighted moving average with a Gaussian kernel [35], and Wilson et al. fit a loess curve on the MA plot as in Dudoit et al.'s study, but then adjust it by smoothing residuals spatially on the array [36]. Various other methods have also been published [37, 38]. Some methods have been proposed in the case of *in situ* oligonucleotide arrays as well, although none is commonly used. Reimers and Weinstein proposed to visualize spatial biases by computing the deviation of each probe intensity from a robust average of that probe's intensity across all arrays in the experiment and plotting these values on the array surface [10]; Suárez-Fariñas et al. used these to identify array regions to discard [11]. Upton and Lloyd propose to subtract the smallest intensity around each probe on the array surface [12]. Arteaga-Salas et al. essentially combine the ideas of Reimers and Weinstein and those of Upton and Lloyd to come up with an algorithm: subtract, from each probe intensity, the average local deviation from the average intensity across replicate arrays, that is, an estimate of locally induced, array-specific error [13]. Various other methods have also been published [14], some of which are applicable only to specific platforms, such as CGH arrays [39] and SNP arrays [40].

The currently advocated standard operating procedure with respect to the well-known issue of spatial bias in *in situ* oligonucleotide array data, when one is used at all, consists of performing quality control steps to identify arrays deemed to be beyond arbitrary acceptability thresholds, and discarding these while leaving others intact [10]. While this may appear to reduce noise on affected probes, it also silently increases noise globally by decreasing replication, and there is comparatively little information on any given gene to begin with [41]. Discarding array regions or even individual probes [10, 11, 42] may alleviate this issue somewhat, though this merely shifts the issue to a lower level. Several studies have concluded that most, if not all, arrays are affected by spatial bias, regardless of platform [14]. We believe that very few of these are likely to be truly unrecoverable; thus, we propose to correct all arrays without prejudice.

We posit that *a priori* information about sources of systematic variation, in the form of known relationships between probes, can be exploited to identify, quantify, visualize, and effectively correct probe-level systematic errors. Here, we present an algorithm, *pyn*, to correct for sources of obscuring variation dependent on spatial location of probes on the array. The algorithm works by leveraging the power of expected mutual information found in probe sets with that of unexpected mutual information found in spatially proximate probes.

## 2. Methods

### 2.1. Algorithm

Using notation inspired by [22], let *Y*_*ijn*_ be the log_2_-transformed HSI of probe *j* = 1,…, *J*_*n*_ belonging to probe set *n* = 1,…, *N*, on array *i* = 1,…, *I*. Error estimates for observed intensities can then be expressed as deviation from some given intensity estimator

(1) $$\begin{array}{c} R_{ijn}\;=Y_{ijn}-{\hat{Y}}_{in}, \end{array}$$

and we propose the following estimator:

(2) $$\begin{array}{c} {\hat{Y}}_{in}=\frac{1}{J_{n}}\sum_{j}Y_{ijn}. \end{array}$$

The probe residual, *R*_*ijn*_, is thus simply the deviation of each HSI from the mean observed in its probe set on the same array.

In justifying the use of spike-in and dilution datasets for assessing accuracy, Cope et al. assert that “to estimate bias in measurements, we need truth, in an absolute or relative form, or at least a different, more accurate measurement of the same samples” [6]. We propose two extensions to this view: first, *a priori* information about relationships between probes provides a form of relative truth; and second, this bias estimate is better used as a correction term than as an accuracy assessment.

Although a given probe residual merely quantifies one HSI's deviation from an estimate and thus contains contributions from many probe-specific biases (e.g., binding efficiency and specificity) and random noise, a sufficiently large pool of probe residuals with similar locations on the array provides a summary of the average bias induced by such a location. Noting that residuals (and accordingly means of residuals) share units and scale with HSIs, we thus simply propose to subtract this estimate of location-induced bias from each HSI to obtain corrected signals:

(3) $$\begin{array}{c} Y_{ijn}^{*}=Y_{ijn}-{\hat{R}}_{ijn}, \end{array}$$

where

(4) $$\begin{array}{c} {\hat{R}}_{ijn}=\frac{1}{k}\sum_{j^{\prime }n^{\prime }\in kNN(jn)}R_{ij^{\prime }n^{\prime }}, \end{array}$$

and *kNN*(*jn*) returns the *k* nearest neighbours of probe *jn*, physically on the array, in terms of Euclidian distance.

It is of theoretical interest to note that, as *k* increases, the computed estimates of local bias tend to approach a constant as they become less local and approach a global measure on the entire array; in the limiting case, where *k* → ∑_*n*_ *J*_*n*_, the correction factor is almost exactly zero. Of practical interest is that this is also the expected behaviour in the case of an ideal array (lacking spatial bias) with a reasonable *k*.

The optimal choice of *k*, the only free parameter of our algorithm, is explored in the *Results *section. When a value is not given in the text, *k* = 20 is to be assumed.

### 2.2. Data

In this paper, three typical Affymetrix Human Genome U133A datasets are used to evaluate the proposed algorithm: two datasets obtained from the public microarray repository GEO [2] and a well-known benchmark dataset; these are referred to in the text as “GSE1400,” “GSE2189,” and “spike-in,” respectively. The GSE1400 experiment compares two samples, with three replicates each: RNA associated with membrane-bound polysomes and RNA associated with free polysomes [44]. The GSE2189 experiment assesses the impact of the chemotherapeutic drug motexafin gadolinium relative to an untreated control sample, at three time points, with three replicates for each of these six samples [45]. In the spike-in experiment, 42 transcripts are spiked in at 14 different concentrations, arranged in a Latin Square design across 14 arrays, such that each transcript is spiked in once at each concentration, with three replicates for each array [46]. For practical reasons, some results present data for only one array per dataset; in these cases, the arrays in question are GSM23121, GSM39803, and Expt1_R1, respectively.

### 2.3. Implementation

An implementation of the proposed spatial correction procedure is provided as part of an R package (“pyn”), with critical parts written in C++ for efficiency. An “AffyBatch” object (“batch”) can be replaced with a corrected version by running the following command within an R session:

> batch <- normalize. pyn (batch)

The procedure runs for approximately one second per array (Intel Core 2 Duo 3.33 GHz) and accepts any valid AffyBatch object, independent of array platform. The user can then proceed with his usual analysis pipeline, for example, “rma” and “limma.” The package also contains some utility functions for generating assessment figures similar to those found in this paper; users are referred to the package's internal documentation for instructions. The package is released under a BSD license and is available at http://lemieux.iric.ca/pyn/.

### 2.4. Alternative Methods

In this paper, “CPP” and “LPE” refer to the two algorithms proposed by Arteaga-Salas et al. in [13]; implementations provided by Dr. Arteaga-Salas were used as they are, with default parameters. “Upton-Lloyd” refers to the method proposed in [12]; as code was not made available by its authors, we used our own implementation, which is provided for convenience in the R package made available with this paper, as the function “upton.lloyd.2005.” We now briefly describe these three algorithms.

#### 2.4.1. LPE

For each probe on each array in a batch, a value similar to our residual is computed, relating the deviation of the probe's intensity from its median intensity across all arrays. A “code” is then computed for each probe, which identifies the array where this value is the largest and whether it is positive or negative. Then, to determine whether a given location in a batch of arrays is affected by spatial bias, PM and MM probes in a 5 × 5 window are inspected; if an “unusual” number of these exhibit the same code, the location on the array identified by this code is flagged for correction. Finally, for each flagged location, a standardized average of the previously defined deviations within its window is subtracted.

#### 2.4.2. CPP

Residuals are computed for each probe on each array as in LPE, but the authors require in this case for all arrays to be replicates of one another. Each PM intensity is corrected by subtracting its MM counterpart's residual and vice versa. Residuals are scaled before subtraction to address differences in PM and MM distributions.

#### 2.4.3. Upton-Lloyd

From each intensity at a given location on an array, the smallest intensity found in a (2*m* + 1) × (2*m* + 1) window is centred on it is subtracted; if the result is negative, it is replaced with zero. The authors find that *m* = 1 works best.

## 3. Results

Figure 1 shows the empirical distribution of probe residuals in each of the 66 arrays found in the three datasets described in the Data subsection. In all cases, the residuals appear to follow an approximately normal distribution with sample mean near zero (|*μ* | <10^−17^). The parameters of this distribution vary from one array to another, based on their intensity distributions, which notably vary in offset (“background intensity”) and scale (“dynamic range”); however, arrays in a common “batch” usually share these parameters. Thus, identifying outliers in residual distributions within a batch may be useful in identifying arrays significantly affected by spatial bias.

Under the assumption that probes are randomly located on the array, these residuals are expected to be randomly spatially distributed across the array; thus, any spatial patterns must be ascribable to some form of technical error. In practice, we frequently observe various manifestations of such patterns.  Figure 2(a) plots the empirical spatial distribution of probe residuals for three arrays as heat maps, allowing for visual, qualitative assessment. GSE1400 is a typical case, in which the only noticeable regularities perceived are a few array-wide, horizontal stripes showing a greater density of either positive or negative residuals; GSE2189 presents a more problematic case, where a large region of the array (left side) exhibits a large cluster of positive residuals; finally, the spike-in dataset reveals similar stripes as in GSE1400, in addition to a small localized artefact showing exclusively negative residuals (bottom right).

The spatial patterns identified in Figure 2 are reminiscent of those previously identified by plotting each probe intensity's deviation from an arbitrary reference array [47], from the average of that probe's intensity in replicate arrays [10], in all arrays in the experiment [11, 13], or in all arrays found in GEO [14], and by plotting the residuals (or some variation thereof) of a probe-level model-based method [48, 49]. However, previous work has been limited to qualitative and subjective quality control purposes, with few exceptions to the best of the authors' knowledge [12, 13]. We propose to exploit these values in a background correction algorithm. Moreover, our definition of probe residuals, not based on other arrays, allows for the indiscriminate identification of both systematic (e.g., batch effects, scanner effects) and array-specific spatial biases (e.g., sample spatial nonuniformity, smudges, scratches).

Figure 2(b) shows the spatial distribution of probe residuals on the three arrays after correction by *pyn*. Qualitatively, all three types of artefacts previously identified appear to have been eliminated or at least severely reduced, thereby marginalizing spatial dependence between residuals.

This “spatial dependence” can be defined quantitatively as *spatial autocorrelation*, the bidimensional extension to autocorrelation, which itself is a special case of correlation in which the two vectors under analysis *u* and *v* are such that *u*_*i*_ = *v*_*i*+1_ for *i* = 1, …, | *u*|. This additional dimensionality and directionality leads to multiple alternative formulations of a quantitative metric: Geary's C [50] and Moran's I [51] are widely used in geostatistics, while Reimers and Weinstein [10] and Wilson et al. [36] have proposed metrics specifically for microarrays. Though we have implemented all four of these metrics, results are only shown for Reimers-Weinstein as all results were found to be comparable (data not shown).  Figure 3 assesses the impact of correcting each of the 66 arrays in our study with the four methods described in the Methods section. CPP and LPE leave all arrays largely unchanged, with the exception of GSM38903 (the selected array from GSE2189, with large blob in Figure 2(a)); in all cases, Upton-Lloyd results in the lowest spatial autocorrelation (near zero), while *pyn* results in the second lowest.

As effective correction of array-specific spatial biases should result in greater reproducibility, we evaluated the impact of each spatial correction method on variance across replicate arrays.  Figure 4 shows the standard deviation across replicate arrays of gene expression values as obtained by RMA after pretreatment by each of the methods, as a function of mean log expression value. At low expression levels, *pyn* performs best, Upton-Lloyd critically inflates standard deviation, and CPP and LPE appear to have no effect whatsoever; at higher expression levels, all methods appear to have virtually no effect.

In order to assess the impact of the spatial correction methods on more tangible, biological results, we used the *affycomp* package [43] to assess differentially expressed gene (DEG) detection power in a dataset in which DEGs are “known.” The tool separates genes spiked in at low (Figure 5(a)), medium (Figure 5(b)), and high concentrations (Figure 5(c)); in each plot, the *x*-axis conveys 1 − specificity, while the *y*-axis conveys sensitivity (see [6, 43] for details). *pyn* significantly improves low- and medium-concentration DEG detection power, while Upton-Lloyd clearly deteriorates it, and CPP and LPE appear to have virtually no effect; at high concentrations, no method can improve DEG detection power, though Upton-Lloyd and CPP deteriorate it significantly.

Finally, we assessed the effect of parameter *k*, the number of neighbouring residuals pooled in (3). ROC curves are generated before correction and after correction with varying values of *k*, and the area under the curve (AUC) is computed for each ROC curve.  Figure 6 shows the difference between the AUC in corrected data and the AUC in original data (ΔAUC), as a function of *k*, separately for low-, medium-, and high-concentration spike-in genes. Small values of *k* produce less robust (noisy) estimates, as they are more susceptible to contain contributions from other sources of probe-specific, but not location-dependent, variations; thus, as expected, small values of *k*(<6) perform badly. Conversely, as *k* increases, estimates of local bias, that is, the ${\hat{R}}_{ijn}$'s, become increasingly robust; thus, low- and medium-concentration DEG detection power is increased significantly above this threshold, peaking around *k* = 20 then levelling off (not shown, convergence to ΔAUC = 0). Above the *k* = 6 threshold, high-concentration DEG detection power is unaffected. As explained in the Algorithm subsection, as *k* continues to increase, the correction gradually becomes a simple scaling of all intensities by a constant; thus, the curves in Figure 6 would approach zero as *k* continues to increase (data not shown).

## 4. Discussion

Systematic, nonbiological variations have been long known to obscure microarray data. In the case of spotted arrays, array- and print-tip-dependent biases were the first to be considered. Array bias could trivially be visualized by inspecting box plots of a batch of arrays or using more sophisticated approaches such as RLE and NUSE plots [33]; correction of such biases usually consisted of subtracting each array's mean or median intensity to impose a common average on each array's HSI distribution [33]. Print-tip bias could similarly be visualized by inspecting intensity distributions for each print tip, plotted side by side; a proposed correction consisted of subtracting a loess fit of this plot for each array [25, 26]. Intensity-dependent bias can be visualized and corrected for in the same manner, in which case the plots are known as MA plots and the correction is that first proposed in [25].

This strategy of addressing known sources of bias individually has been somewhat abandoned in the case of *in situ* oligonucleotide arrays, perhaps based on the assumption that streamlined commercial manufacturing of such high-density arrays is much less prone to significant systematic errors. For example, background correction and normalization steps in two of the most popular analysis packages, RMA [22] and GCRMA [52], do not take into account known sources of bias, but rather make global, sweeping transformations of the data, via a convolutional model [53] and quantile normalization [27], respectively. 

We posit, as initially asserted by Dudoit et al. in 2002, that correcting for known biases using *a priori* information is preferable to global, blind, generic normalization, and/or reliance on unverified modelling assumptions [26]. We have thus proposed such a scheme for correcting spatial bias in Affymetrix GeneChip data, which can readily be applied on other platforms using multiple probes per gene.

We have shown that this method reduces spatial autocorrelation in HSIs, reduces variance in gene expression measures across replicate arrays, improves DEG detection power, and performs better than previously published methods in terms of replicate variance reduction and DEG detection power increase. As for spatial autocorrelation reduction, we conclude that Upton-Lloyd removes “too much” due to its working directly on HSIs as opposed to *pyn*'s working with residuals, which may avoid subtracting the “baseline” spatial autocorrelation inherent to each microarray platform.

Our analysis of parameter *k*, the number of neighbouring residuals pooled in (3), identified *k* = 6 as a minimum in order for correction to improve DEG detection power and *k* = 19 as the optimal value. As computational time is linearly proportional to *k* and values in the 12 ≤ *k* ≤ 30 range all result in comparable performance, we propose to compromise with *k* = 20, and this is the default in the provided implementation.

An assessment based on a spike-in benchmark dataset indicated that DEG detection power is increased for low- and medium-concentration genes and is insignificantly affected for high-concentration genes. However, it should be noted that, by its very design, this central *affycomp* assessment does not take into account methods' ability to increase detection power *across replicate arrays*, that is, to take array-specific effects and so-called “batch effects” into consideration: a ROC curve is computed for each possible pair of arrays *within each of the three 14-array batches*, and the 3 × *C*(14,2) = 3 × 91 = 273 curves are averaged to generate plots such as Figure 5 [6, 43]. As the great majority of microarray experiments feature more than one replicate array per sample, this setup is highly unrepresentative of real conditions. Additionally, it should be noted that the spike-in dataset is at the high end of the quality control spectrum; thus, if our correction method is able to improve biological results resulting from this data, it is to be expected that improvement will be even more significant for everyday datasets, such as GSE1400 and GSE2189.

 Although the “random” spatial distribution of probes on the array surface was presented in this paper as a necessary assumption, this is an oversimplification and, thus, not strictly correct. In reality, the underlying assumption is that probe locations are independent of their targets, or—in more practical terms—of the locations of probes in the same probe set, such that any correlation between locations and residuals is always considered undesirable noise or bias; this can also be expressed as the assumption that probes are randomly spatially distributed on the array surface *within each probe set*. Thus, although recent studies have uncovered a significant dependence between probe locations and their sequences by observing spatial patterns when spatially mapping probe sequence GC content on the array [10, 14], our algorithm's assumptions are unaffected. This may also explain why, although Upton-Lloyd appears to reduce spatial autocorrelation further than our method, it performs much worse in replicate array variance and DEG detection power. In addition, these results imply that spatial bias correction procedures may unintentionally (likely only partially) correct sequence biases as well; conversely, it implies that sequence bias correction may somewhat correct spatial biases in the process as well. This does not appear to be problematic *per se*, but is likely worthy of further investigation in order to fully understand its implications.

 Finally, the framework established herein provides opportunity for implementing further types of microarray data pretreatments: correction of a specific source of bias which can be expressed as the presence of undesirable mutual information shared by “neighbouring” probes in some given coordinate space (e.g., physical location on the array) or based on some given distance metric, as opposed to expected, desirable mutual information shared by some other sets of probes (e.g., Affymetrix “probe sets”). This approach (which we dub *pyn*: *probe your neighbours*) should be applicable to other biases such as probe composition as well as to other microarray platforms such as tiling arrays, and preliminary results indicate that this is indeed the case.

## 5. Conclusions

Oligonucleotide array data is invariably biased by a number of confounding factors, some of which can be effectively quantified and eliminated. We have proposed a method for correcting bias arising from a known source and show the efficacy of one case, namely, spatial bias in Affymetrix GeneChip data. An implementation is provided as a convenient R package, released under the BSD license, available at http://lemieux.iric.ca/pyn/.

## Figures and Tables

**Figure 1:**
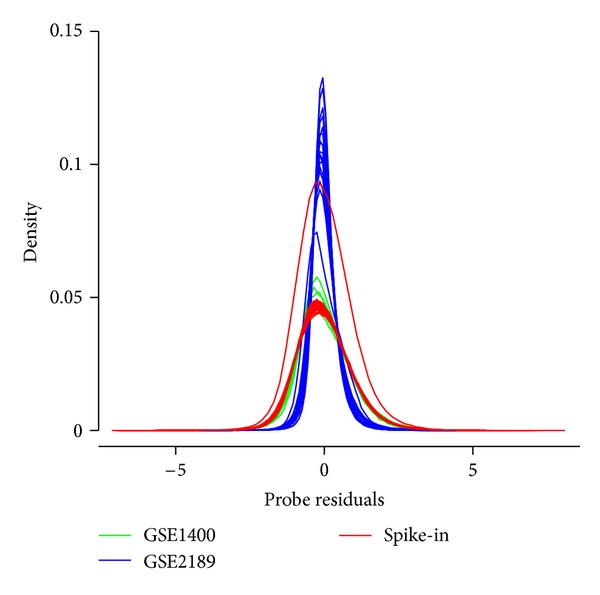
Empirical distributions of probe residuals R, as defined in the text, with one curve associated with each of the 66 arrays found in the three datasets. In each case, the mean residual is almost zero (|*μ* | <10^−17^).

**Figure 2:**
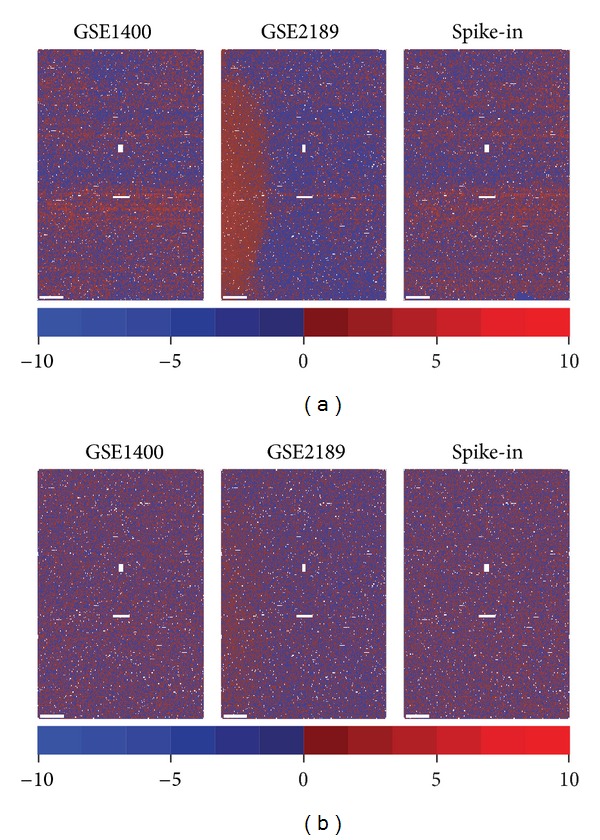
Empirical spatial distribution of probe residuals in original and corrected data. Mapping probe residuals back to their originating physical locations and displaying them as a heat map reveals a variety of spatial artefacts in (a) original data: horizontal stripes (all arrays), a large region of positive residuals (GSE2189, left), and a small region of negative residuals (spike-in, bottom right); (b) after correction (*k* = 20), all of these artefacts appear to have been eliminated or greatly attenuated. Non-PM locations (MM and control probes) are coloured in white.

**Figure 3:**
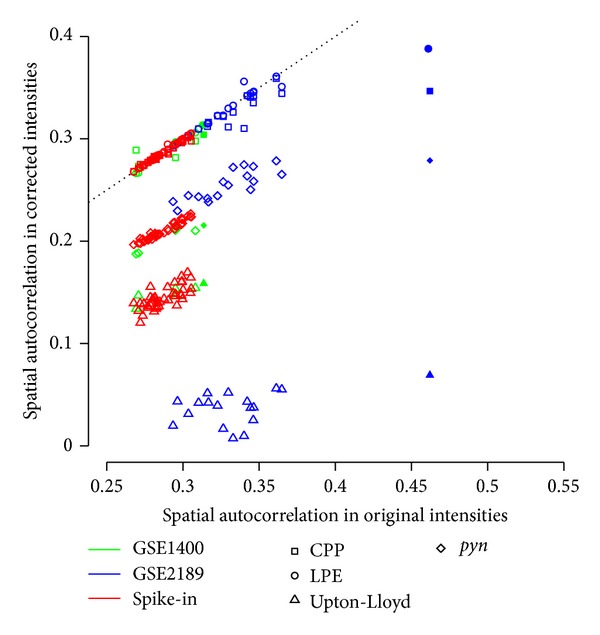
Quantitative effects of correction on spatial autocorrelation. Reimers-Weinstein spatial autocorrelation metric computed in data corrected by various methods (*y*-axis) and in original data (*x*-axis) for each array in each dataset, with the “selected” array from each dataset being emphasized by a solid bullet. The Reimers-Weinstein metric is a Pearson correlation coefficient computed between each intensity and the average intensity among its four neighbours on the array [10]. An unchanged metric lies on the dotted unit line, while a value below (above) this line indicates a decrease (increase) in spatial autocorrelation. Upton-Lloyd consistently results in the greatest decrease, followed by *pyn*, while CPP and LPE have no effect in all but one case.

**Figure 4:**
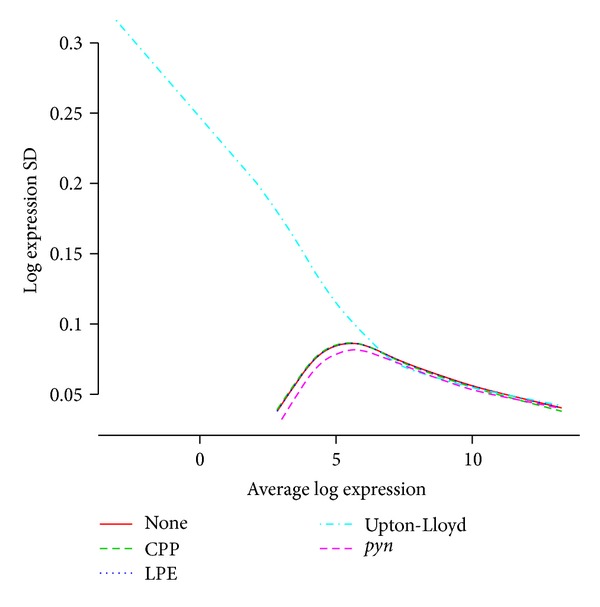
Effect of correction on reproducibility across replicate arrays. Standard deviation of log expression index of probe sets across replicate arrays as a function of mean log expression, as computed in data obtained with RMA (all default parameters) after pretreatment with each of the spatial correction methods (or none). *pyn* performs best, resulting in an increase in reproducibility notably for low-expression genes, while Upton-Lloyd deteriorates data for low-expression genes, and CPP and LPE have virtually no effect.

**Figure 5:**
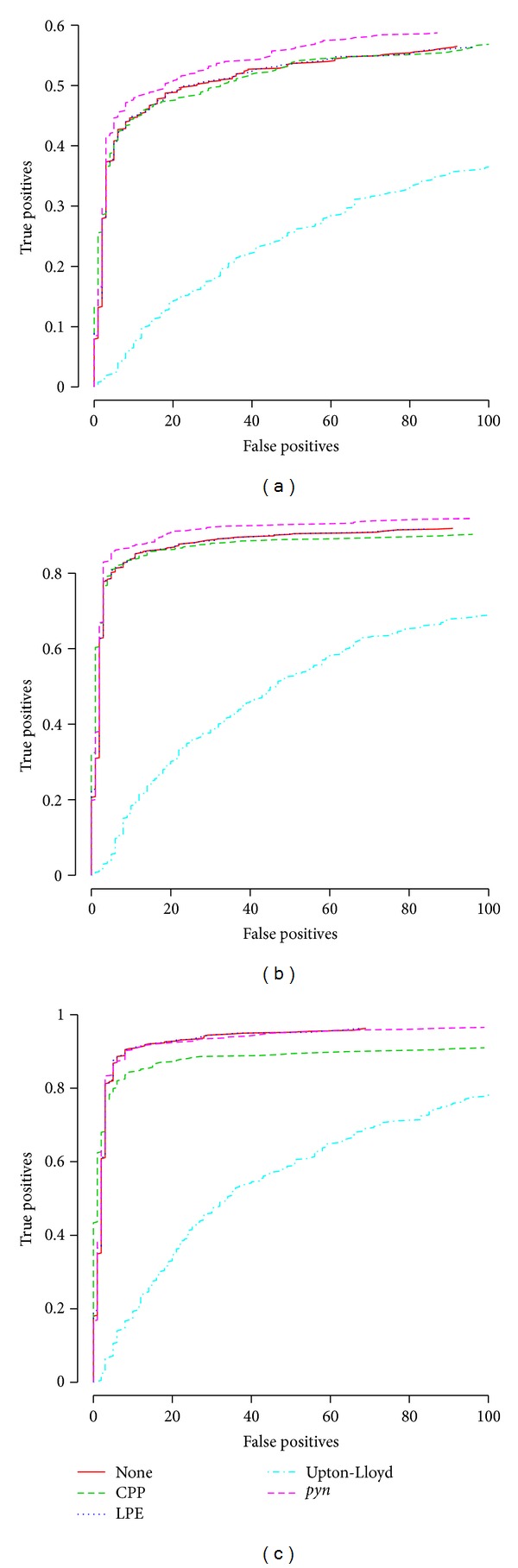
Effect of correction on DEG detection power. Receiver operating characteristic (ROC) curves generated by the R/bioconductor package *affycomp*, plotting *sensitivity* as a function of (1 – *specificity*), as computed in data obtained with RMA (all default parameters) after pretreatment with each of the spatial correction methods (or none) for (a) low-, (b) medium-, and (c) high-concentration spike-in genes. See [6, 43] for details. At low and medium concentrations, *pyn* performs the best; at all concentrations, Upton-Lloyd performs the worst by a large margin; CPP and LPE have virtually no effect, with the exception of CPP degrading results at high concentration.

**Figure 6:**
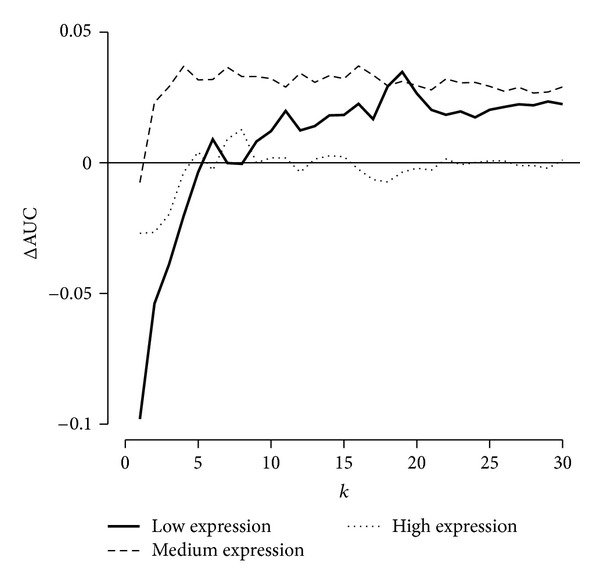
Effect of *k* on DEG detection power. Difference between area under the curve (AUC) of receiver operating characteristic (ROC) curves computed in corrected data and AUC computed in original data, as a function of the value used for *k*, separately for low-, medium, and high-concentration spike-in genes. *pyn* improves low- and medium-concentration DEG detection power with *k* > 5, with optimal performance at *k* = 19, and has virtually no effect on high-concentration DEG detection power above *k* = 5.
